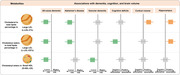# Circulating metabolites and risk of incident dementia: A prospective cohort study

**DOI:** 10.1002/alz.084495

**Published:** 2025-01-03

**Authors:** Shu‐Yi Huang

**Affiliations:** ^1^ Huashan Hospital, Fudan University, Shanghai China

## Abstract

**Background:**

Identifying circulating metabolites associated with dementia, cognition, and brain volume may improve the understanding of dementia pathogenesis and provide novel insights for preventive and therapeutic interventions.

**Method:**

This cohort study included a total of 87,885 participants (median follow‐up 9.1 years, 54% female) without dementia at baseline from the UK Biobank. 249 plasma metabolites were measured using nuclear magnetic resonance spectroscopy at baseline. Cox proportional regression was used to examine the associations of each metabolite with incident dementia (cases = 1134), Alzheimer’s disease (AD; cases = 488), and vascular dementia (VD; cases = 257) during follow‐up. Dementia‐associated metabolites were further analyzed for association with cognitive deficits (N = 87,885) and brain volume (N = 7756) using logistic regression and linear regression.

**Result:**

We identified 26 metabolites associated with incident dementia, of which six were associated with incident AD and five were associated with incident VD. These 26 dementia‐related metabolites were subfractions of intermediate‐density lipoprotein, large low‐density lipoprotein (L‐LDL), small high‐density lipoprotein (S‐HDL), very‐low‐density lipoprotein, fatty acids, ketone bodies, citrate, glucose, and valine. Among them, the cholesterol percentage in L‐LDL (L‐LDL‐C%) was associated with lower risk of AD (HR[95%CI] = 0.92[0.87‐0.97], p = 0.002), higher brain cortical (β = 0.047, p = 3.91 × 10^‐6^) and hippocampal (β = 0.043, p = 1.93 × 10^‐4^) volume. Cholesteryl ester to total lipid ratio in L‐LDL (L‐LDL‐CE%) was associated with lower risk of AD (HR[95%CI] = 0.93[0.90‐0.96], p = 1.48 × 10^‐4^), cognitive deficits (odds ratio = 0.98, p = 0.009), and higher hippocampal volume (β = 0.027, p = 0.009). Cholesteryl esters in S‐HDL (S‐HDL‐CE) were associated with lower risk of VD (HR[95%CI] = 0.81[0.71‐0.93], p = 0.002), but not AD.

**Conclusion:**

Taken together, circulating levels of L‐LDL‐CE% and L‐LDL‐C% were robustly linked to risk of AD and AD phenotypes, but not with VD. S‐HDL‐CE was associate with lower risk of VD, but not with AD or AD phenotypes. These metabolites may play a role in the advancement of future intervention trials. Additional research is necessary to gain a complete comprehension of the molecular mechanisms behind these associations.